# Identification of subgroups and development of prognostic risk models along the glycolysis–cholesterol synthesis axis in lung adenocarcinoma

**DOI:** 10.1038/s41598-024-64602-7

**Published:** 2024-06-26

**Authors:** Jiuzhou Jiang, Bao Qian, Yangjie Guo, Zhengfu He

**Affiliations:** 1 Department of Thoracic Surgery, Sir Run Run Shaw Hospital, Medical College of Zhejiang University, Hangzhou, China; 2 Zhejiang University School of Medicine, Hangzhou, China

**Keywords:** Glycolysis, Cholesterol, XGBoost, SHAP, Cancer metabolism, Lung cancer, Genome informatics

## Abstract

Lung cancer is one of the most dangerous malignant tumors affecting human health. Lung adenocarcinoma (LUAD) is the most common subtype of lung cancer. Both glycolytic and cholesterogenic pathways play critical roles in metabolic adaptation to cancer. A dataset of 585 LUAD samples was downloaded from The Cancer Genome Atlas database. We obtained co-expressed glycolysis and cholesterogenesis genes by selecting and clustering genes from Molecular Signatures Database v7.5. We compared the prognosis of different subtypes and identified differentially expressed genes between subtypes. Predictive outcome events were modeled using machine learning, and the top 9 most important prognostic genes were selected by Shapley additive explanation analysis. A risk score model was built based on multivariate Cox analysis. LUAD patients were categorized into four metabolic subgroups: cholesterogenic, glycolytic, quiescent, and mixed. The worst prognosis was the mixed subtype. The prognostic model had great predictive performance in the test set. Patients with LUAD were effectively typed by glycolytic and cholesterogenic genes and were identified as having the worst prognosis in the glycolytic and cholesterogenic enriched gene groups. The prognostic model can provide an essential basis for clinicians to predict clinical outcomes for patients. The model was robust on the training and test datasets and had a great predictive performance.

## Introduction

Although lung cancer is the second most common cancer globally, it is the leading cause of cancer deaths, accounting for an annual estimated total of two million new cases and 1.76 million deaths^1,2^. Lung cancer can be broadly grouped into small-cell lung cancer (SCLC, 15%) and non-small-cell lung cancer (NSCLC, 85%), and lung adenocarcinoma (LUAD) is the most common subtype of NSCLC^2–4^. The treatment of NSCLC has changed dramatically over the past decade, primarily due to advances in biomarkers that allow for targeted and immune-based therapies for specific patients with significant success^5^. However, the vast majority of advanced NSCLC become resistant to current treatments and eventually progress^6^. Therefore, searching for new predictors to predict and improve the prognosis of LUAD is imminent.

Reprogramming of cell metabolism is an essential feature of malignancy, as shown by abnormal uptake of glucose and amino acids and dysregulation of glycolysis^7,8^. Glycolysis is a specific metabolic pattern of tumor cells, which meets the requirements of tumor cells for ATP, etc^9^. Mitochondrial pyruvate carrier (MPC) consists of MPC1, and MPC2 is responsible for the import of pyruvate from the cytoplasmic matrix into the mitochondrial matrix, which can affect glycolysis, and damaged MPC function may induce tumors with solid capabilities for proliferation, migration, and invasion^10,11^. Pyruvate is converted to acetyl coenzyme A, which is further changed to citric acid, a precursor substance required for lipogenesis, including the synthesis of cholesterol^12^. There is growing evidence for a close relationship between cholesterol metabolism and some types of cancer, such as allosteric interactions in the microenvironment of tumors, cancer cell spreading and metastasis forming, and lipid metabolism in tumor-initiating cells (TICs)^13,14^.

In recent decades, some studies have investigated potential prognostic signatures of LUAD using only bulk RNA- seq data, which principally provides data on the average of the total number of cells in the sample^15,16^. Single-cell sequencing is a powerful instrument for dissecting the cellular and molecular landscape with single-cell resolution, revolutionizing our comprehension of the biological features and dynamics within cancer pathologies^17^. Single-cell RNA-seq technology can comprehensively characterize the heterogeneity of the tumor microenvironment and help dissect the complex cell type compositions and expressive heterogeneity in TME, and the Tumor Immune Single Cell Hub (TISCH) can assist us with a simple analysis^18^.

The analysis of large volumes of complex biomedical data through computer algorithms, driven by the ongoing development of computer hardware and enormous amounts of data, offers substantial advantages for advancing biology and accurately estimating patient conditions^19,20^. Machine learning (ML) is a scientific discipline focusing on how computers learn from data and build predictive models. It is becoming an embedded part of modern research in biology, but its “black box” nature is an additional challenge^21,22^. Many algorithms are widely used to analyze complex biomedical data, such as extreme gradient boosting (XGBoost)^23^, Random Forest Classifier (RFC)^24^, Logistic Regression (LR)^25^, Support Vector Machine (SVM)^26^, and K-Nearest Neighbors (KNN)^27^. Interpretation is an integral branch of method development, with Shapley additive explanation (SHAP) being an integral approach^28^. The SHAP, which explains the model outcome by computing the contribution of each input feature for all samples, was applied to study the effects of different variables^29,30^. A positively or negatively valued SHAP represents an increase or decrease in the probability of a specific outcome.

Balanced glycolysis and cholesterol production pathways can jointly regulate tumor progression. However, there is a lack of studies to establish LUAD-related staging based on the glycolysis–cholesterol synthesis axis. So based on the gene expression levels of glycolysis–cholesterogenesis, we defined four subtypes of LUAD from a metabolic perspective and further analyzed the characteristics of different subtypes, such as survival time and other clinical features in this study. Then, we identified prognostic genes based on ML, further explored the correlation of the genes with clinical features and tumor microenvironment (TME), and proposed a risk-prognosis model that can be used to formulate treatment options and analyze the prognosis.

## Materials and methods

### Data acquisition and processing

Gene expression data (count matrix), corresponding clinical information, and the somatic mutation profiles for 585 LUAD patients were downloaded from The Cancer Genome Atlas (TCGA) via the UCSC Xena browser (https://xenabrowser.net/). For RNA-seq data, the ‘cpm’ algorithm of the “edgeR” package (V 2.12.0) was used to convert the count data into counts per million reads mapped (CPM), which was used to estimate the level of expression of each gene, before log2 transformations were performed. Based on the mutation data, the “Maftools” package (V 3.38.1) was used to analyze these results. In this study, we kept the expression profiles of the primary solid tumor samples, removed patients with missing survival information, and filtered out patients with less than 30 days of follow-up.

### Identification of metabolic Subtypes

Glycolysis and cholesterol synthesis genes were obtained from Molecular Signatures Database v7.5 in the ‘REACTOME_GLYCOLYSIS’ (n = 72) and ‘REACTOME_CHOLESTEROL_ BIOSYNTHESIS’ (n = 25) gene set. After removing the genes with no expression in all samples, a total of 93 genes were obtained, including 69 glycolysis genes and 24 cholesterol biosynthesis genes. *ConsensusClusterPlus* V1.60.0 was used to cluster these genes. The parameters were as follows: reps = 100, pitem = 0.8, pfeature = 1, distance = “Spearman”, clusterAlg = “hc”, innerLinkage = “ward.D2”, finalLinkage = “ward.D2”. Median expression of z-scores of two co-expressed genes identified four metabolic subgroups. We defined them as quiescent (glycolysis ≤ 0, cholesterogenesis ≤ 0), glycolytic (glycolysis > 0, cholesterogenesis ≤ 0), cholesterogenic (glycolysis ≤ 0, cholesterogenesis > 0) and mixed (glycolysis > 0, cholesterogenesis > 0)^31^. To determine whether patients in these four types differed from each other, we performed principal component analysis (PCA). Survival analyses were then conducted, and log-rank test *P* values were also analyzed.

### Analysis of MPC1/2 expression

By calculating Spearman correlation coefficients and corresponding false discovery rate (FDR) values (Beyer-Hardwick method) for MPC1/2 and other genes, we identified genes positively and negatively associated with MPC1/2 expression. 1359 and 2026 genes were found to be related positively and negatively to MPC1/2 expression, respectively (Spearman correlation, FDR < 0.05). To explore the possible functions and pathways of the identified positively and negatively associated genes, gene ontology (GO) analysis was conducted by applying the clusterProfiler package^32^. FDR < 0.05 was chosen as the criterion for cutoff.

### Analysis of differential expression genes

Based on the survival analysis of the four subtypes, the two subtypes with the best and worst prognosis were selected for differential expression analysis using limma with threshold: FDR < 0.05 and log2|FC|> 1.5. GO, and Kyoto Encyclopedia of Genes and Genomes (KEGG) enrichment analysis was then performed on selected differentially expressed genes (DEGs), showing the results with FDR < 0.05.

### Prognostic gene selection based on machine learning

The TCGA-LUAD samples were randomly divided into training (80%) and test (20%) sets. And a chi-square test was performed on the training and test samples to show any significant difference between the two sets.

We applied DEGs to construct 5 ML models, such as extreme gradient boosting (XGBoost^23^), Random Forest Classifier (RFC^24^), Logistic Regression (LR^25^), Support Vector Machine (SVM^26^), and K-Nearest Neighbors (KNN^27^) were used to developed prediction models. Stratified k-fold cross-validation was used to validate the performance of the models.

In a predictive classification model, the most fundamental classification evaluation metric is based on comparing predicted and true values. For a binary classifying problem, comparing true and predicted classification results can categorize all samples into four classes: True Positive (TP), True Negative (TN), False Positive (FP), and False Negative (FN). To make the model perform better, a series of manually tuned real experiments were conducted. Various model training algorithms correspond to various hyperparameters, and the best hyperparameters were obtained by cross-validation in the training set.

We used global visualization of SHAP values to show the importance of the input features and selected the top nine most important features as the prognostic genes with the help of the best model. We explored the relationship between prognostic genes and tumor microenvironment based on Tumor Immune Single Cell Hub Database (TISCH).

### Construction and validation of a prognostic risk model

Using multivariate Cox regression analysis to construct a prognostic risk model based on the expression of prognostic genes. Then, we calculated the risk score for each sample as follows:$Risk score = \mathop \sum \limits_{k = 1}^{n} \left( {Coefficient _{k} *expression _{k} } \right)$. The samples were divided into high and low risk groups based on the median value of the risk scores. We validated the robustness of the model by using the training set and the test set and plot the risk score distribution as well as the ROC curve and KM survival curve. The relationship between risk scores and selected clinical characteristics was also analyzed by classifying high and low risk subgroups.

## Results

### Identification of four metabolic subtypes of LUAD based on glycolysis and cholesterol synthesis gene expression

**Figure 1:**
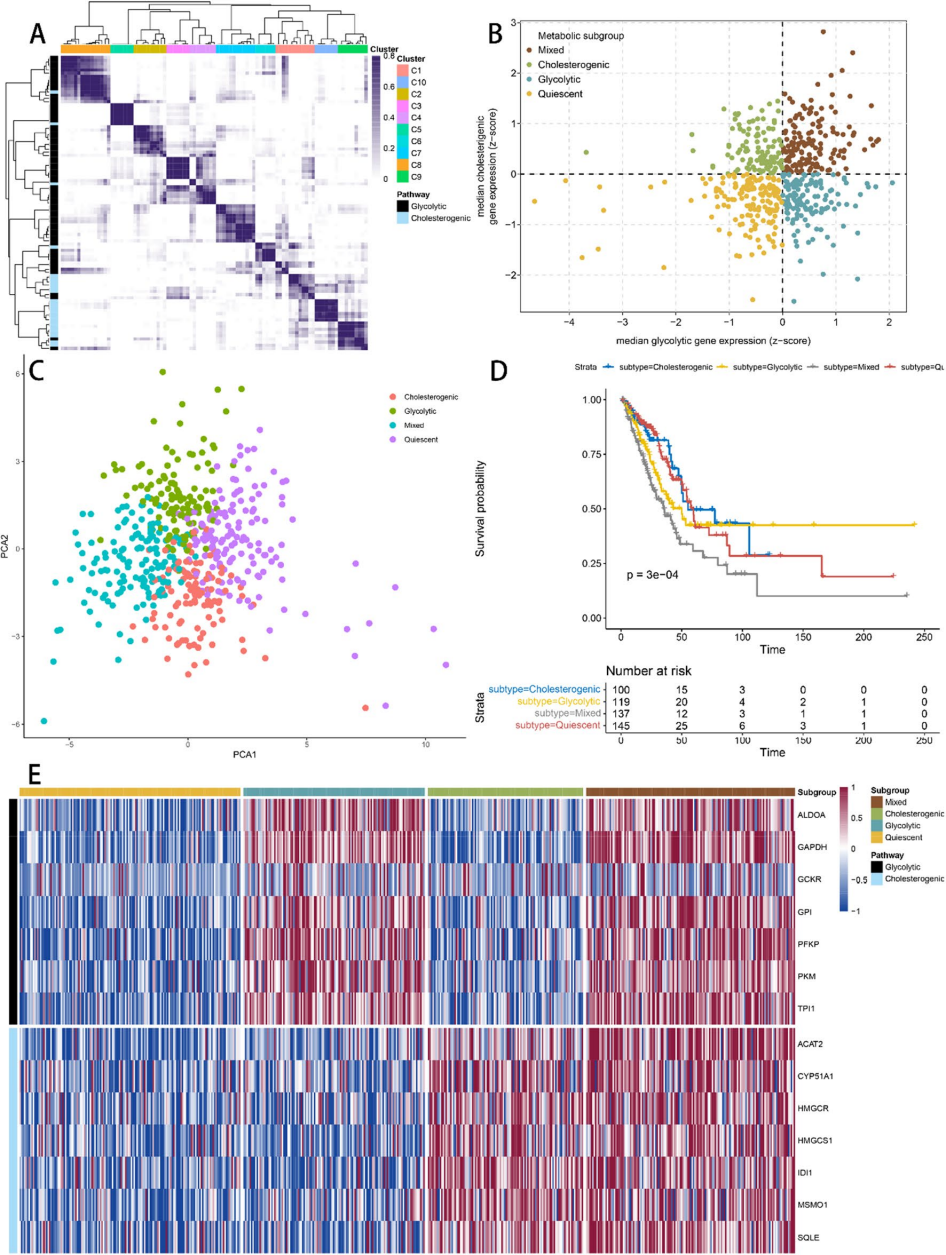
(**A**) Consistent clustering of glycolytic and cholesterogenic genes. (**B**) Sample classification according to median glycolytic and cholesterogenic genes expression. (**C**) Patients with different metabolic subtypes showed significant differences between each other by PCA. (**D**) Kaplan–Meier survival curves for different subgroups of patients. The clinical outcome endpoint was OS. (**E**) Heatmap of expression levels of co-expressed glycolytic and cholesterogenic genes in each metabolic subgroup. PCA, principal components analysis; OS, overall survival; *P*-value of < 0.05 was considered statistically significant.

After screening, 501 LUAD tumor samples from TCGA were used to select co-expressed cholesterogenic and glycolytic genes (Fig. 1A). When k = 10, the genes in C3 were identified as glycolytic co-expression genes, all of which are in the glycolytic pathway (ALDOA, GAPDH, GCKR, GPI, PFKP, PKM, TPI1), and the genes in C10 were identified as cholesterogenic co-expression genes, all of which are in the cholesterogenic pathway (ACAT2, CYP51A1, HMGCR, HMGCS1, IDI1, MSMO1, SQLE). Samples were assigned to four subtypes (Fig. 1B). PCA was performed to demonstrate differences between these subgroups (Fig. 1C). Kaplan–Meier survival curve indicated significant prognostic differences among the four subtypes (log-rank test, *P* = 0.0003) and the worst prognosis for patients in the mixed group and the best in the cholesterogenic group (Fig. 1D). Figure 1E shows the co-expressed cholesterogenic and glycolytic genes expression of the four metabolic subtypes.

### MPC complexes as possible regulators of the glycolysis–cholesterol synthesis axis in tumors

**Figure 2:**
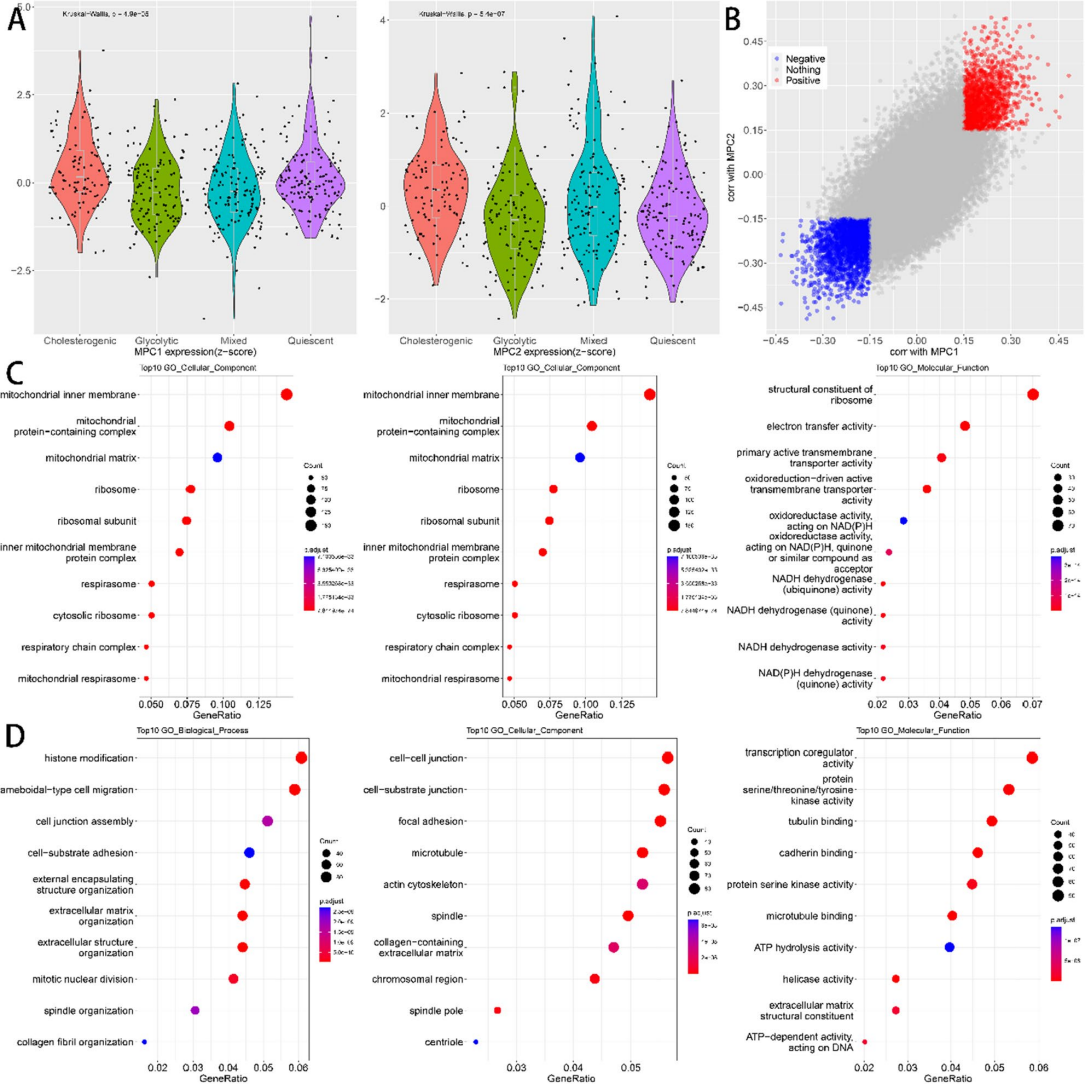
(**A**) MPC1/MPC2 expression across different metabolic subtypes. (**B**) Scatter plot describing the correlation between MPC1 (X-axis) and MPC2 (Y-axis). (**C**) Results of GO enrichment analysis of genes positively associated with MPC1/2. (**D**) Results of GO enrichment analysis of genes negatively associated with MPC1/2.

Among the four metabolic subtypes, the expression of MPC1 and MPC2 was significantly different (Fig. 2A). The mean levels of MPC1 and MPC2 expression were higher in patients in the cholesterogenic group than in the other subtypes, and MPC1 expression was significantly higher in the cholesterogenic group than in the mixed group in particular (FDR < 0.5). Previous studies have shown that MPC1 is absent or underexpressed in a variety of cancers and is associated with poor prognosi^33^. To explore the pathways correlated with MPC1/2 expression levels, we conducted a correlation analysis between MPC1/2 and all the other genes. In total, 1359 and 2026 genes were found to be positively and negatively associated with MPC1/2, respectively (Spearman correlation, BH adjusted *P* < 0.01, Fig. 2B). Further GO functional enrichment analysis showed that genes with positive association with MPC1/2 were implicated in the mitochondrial inner membrane, mitochondrial, protein-containing complex, mitochondrial matrix, and ribosome (Fig. 2C); genes with negative association with MPC1/2 were associated with histone modification, ameboidal-type cell migration, cell junction assembly, cell-substrate adhesion, and external encapsulating (Fig. 2D). MPC1/2 genes are engaged in a cellular network associated with the malignant development of LUAD.

### Differential expression analysis between cholesterogenic and mixed groups

**Figure 3:**
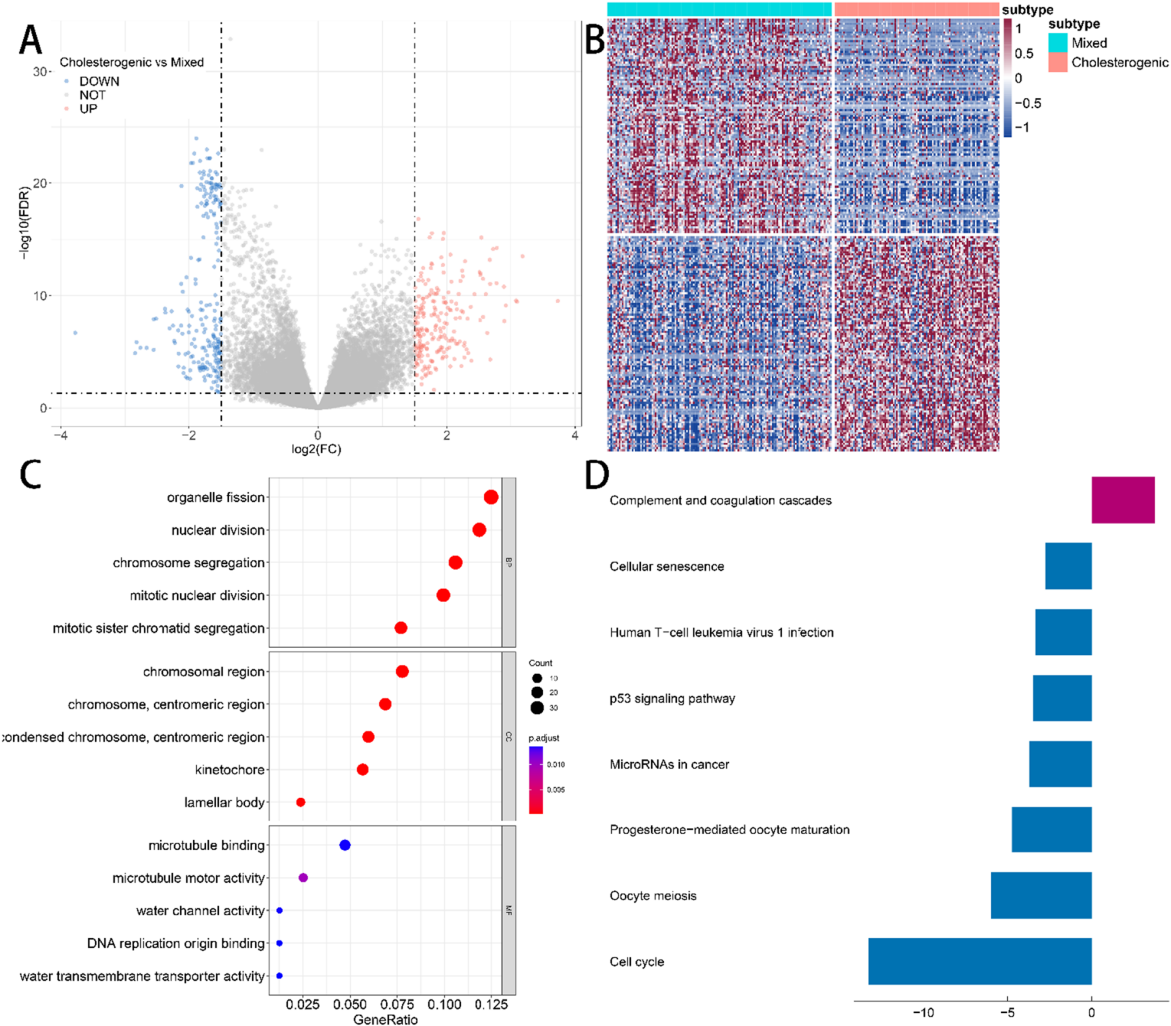
(**A**) Volcano map of differentially expressed genes in the TCGA dataset for cholesterogenic and mixed subtypes. (**B**) Heatmap of differentially expressed genes in the TCGA dataset for cholesterogenic and mixed subtypes. (**C**) Annotation using GO for differentially expressed genes. (**D**) Annotation using KEGG for differentially expressed genes.

The cholesterol subtype had the best prognosis, in contrast to the mixed subtype, which had the worst in the four metabolic subtypes, suggesting that patients with high expression of only cholesterol-related genes had a better outcome, while those with high expression of glycolytic and cholesterol synthesis genes had a worse outcome. To further investigate the effect of cholesterol and resting subtypes on cancer prognosis, 445 DEGs were identified using the *“limma”* package (V3.52.2), of which 221 were up-regulated and 224 were down-regulated (Fig. 3A). Figure 3B shows the heatmap of the 100 genes with the largest up- and down-regulation differences, respectively. GO enrichment analysis and KEGG pathway analysis were performed on 200 DEGs, and the annotated results (FDR < 0.05) are visualized in Fig. 3C–D. The results showed that complement and coagulation cascades, cellular senescence, human T-cell leukemia virus 1 infection, p53 signaling pathway, microRNAs in cancer, and cell cycle are enriched significantly.

### Prognostic gene selection based on machine learning

**Figure 4:**
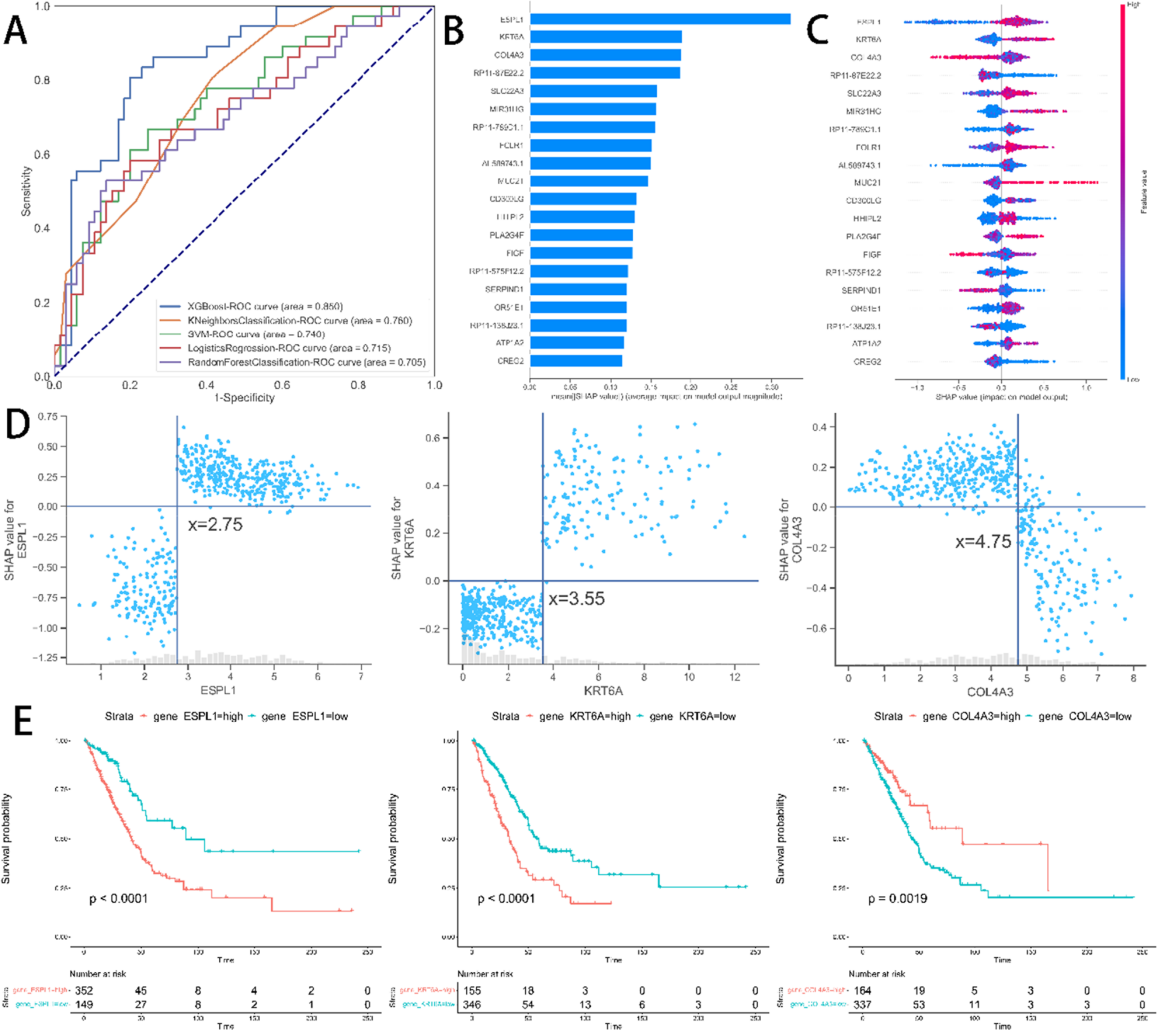
(**A**) Receiver operating characteristic curves showing the performance of the five models. (**B**) Histogram of mean Shapley additivity explained (SHAP) value for the top 20 predictors. (**C**) bee-swarm plots. The X-axis is the SHAP value, representing the impact of the outcome predictors on the Y-axis. One dot represents one individual patient. The higher the SHAP value, the better prognosis. (**D**) The dependence plot of the ESPL1, KRT6A, and COL4A3. (E) Three gene KM curves on the training set.

A total of 501 participants were analyzed, among which 320 were alive; these 501 participants were randomly split into training and test datasets. The clinical characteristics of the study participants are summarized in Table 1. The results of all ML models for predicting survival status are shown in Table 2 and plotted in Fig. 4A. The XGBoost model had the better performance in the test set.

**Table 1:** Clinical characteristic information of the study objects.

Clinical characteristics	Training set	Test set	*P*-value
OS			
0	255	65	1
1	145	36	
Stage			
I	215	58	0.0509
II	101	15	
III	56	23	
IV	20	5	
Age			
< 60	116	20	0.07145
≥ 60	275	80	
Gender			
Female	212	56	0.7424
Male	188	45	

*OS* overall survival.

**Table 2:** Performance classification.

Model	AUC	Accuracy	Sensitivity	Specificity
XGB	0.850	0.792	0.500	0.954
KNN	0.761	0.723	0.278	0.969
SVM	0.740	0.683	0.667	0.692
LR	0.715	0.683	0.222	0.939
RFC	0.705	0.693	0.250	0.939

*AUC* Area under the ROC curve, *XGBoost* Extreme gradient boosting, *KNN* K-nearest neighbors, *SVM* Support vector machine, *LR* Logistic regression, *RFC* Random forest classifier.

In Fig. 4B–C, the summary plot of the mean absolute SHAP values shows the order of importance of the features from highest to lowest with the help of the XGBoost model. It shows that ESPL1, KRT6A, COL4A3, RP11-87E22.2, SLC22A3, MIR31HG, RP11-789C1.1, FOLR1, and AL589743.1 are the nine most important genes for predicting survival status. Figure 4D shows the association between the SHAP values of the three most important genes and the expression of each gene. The truncation thresholds for these three predictor variables can be defined from the graph to distinguish between good prognosis (i.e., SHAP-value < 0) and poor prognosis (i.e., SHAP-value > 0). ESPL1 beyond 2.75 or KRT6A greater than 3.55 or COL4A3 less than 4.75 increases the SHAP value and thus the poorer prognosis. Kaplan–Meier survival curves were plotted for high and low risk groups according to the gene cutoff values, and the results indicate significant prognostic differences between the groups determined by the SHAP values (Fig. 4E).

### Model construction and evaluation

**Figure 5:**
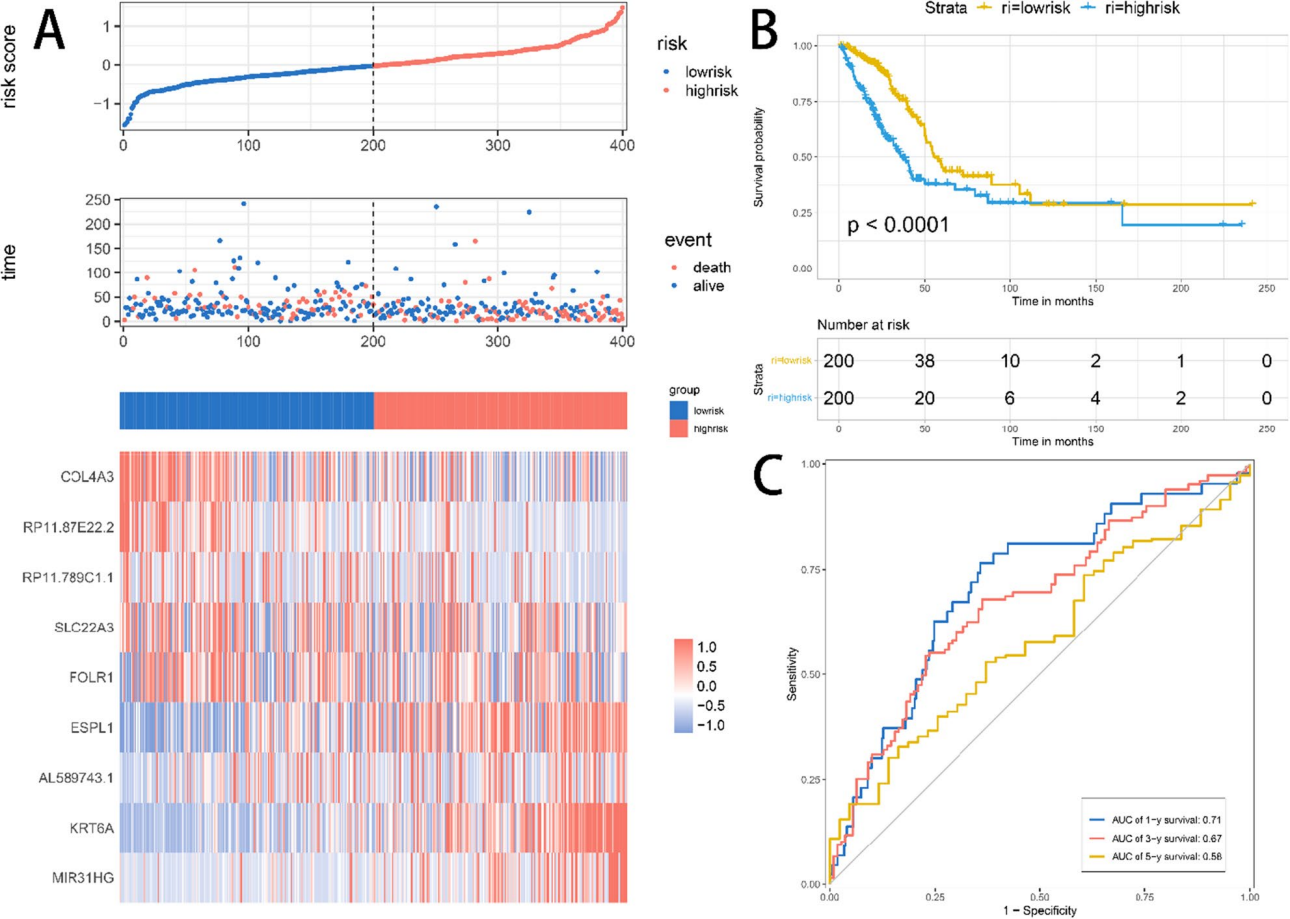
(**A**) Distribution of risk scores, survival time, and heatmap of expression of 9 genes in the training set. (**B**) The KM survival curve of the risk model in the training set. (**C**) The ROC curve of the risk model in the training set.

Using a multivariate cox regression model the nine-gene signature equation is as shown below: risk score** = **1.32*ESPL1 + 1.11*KRT6A + 0.96*COL4A3 + 0.7*RP11-87E22.2 + 1.10*SLC22A3 + 1.22*MIR31HG + 1.08*RP11-789C1.1 + 0.96* AL589743.1 + 1.06*FOLR1. Risk scores were calculated using prognostic gene expression levels for each sample, and risk score distributions and gene expression heatmap of the training set were plotted (Fig. 5A). Survival times were significantly shorter in the high-risk score samples than in the low-risk score samples, suggesting that samples with higher risk scores are more likely to have a poor prognosis. Two hundred samples with risk scores greater than the median were classified as a high-risk group, and 200 samples less than the median were classified as a low-risk group. Significant prognostic differences existed between the high- and low-risk groups (*p* < 0.05, Fig. 5B). Figure 5C shows the predicted classification performance based on risk scores for 1, 3, and 5 years.

**Figure 6:**
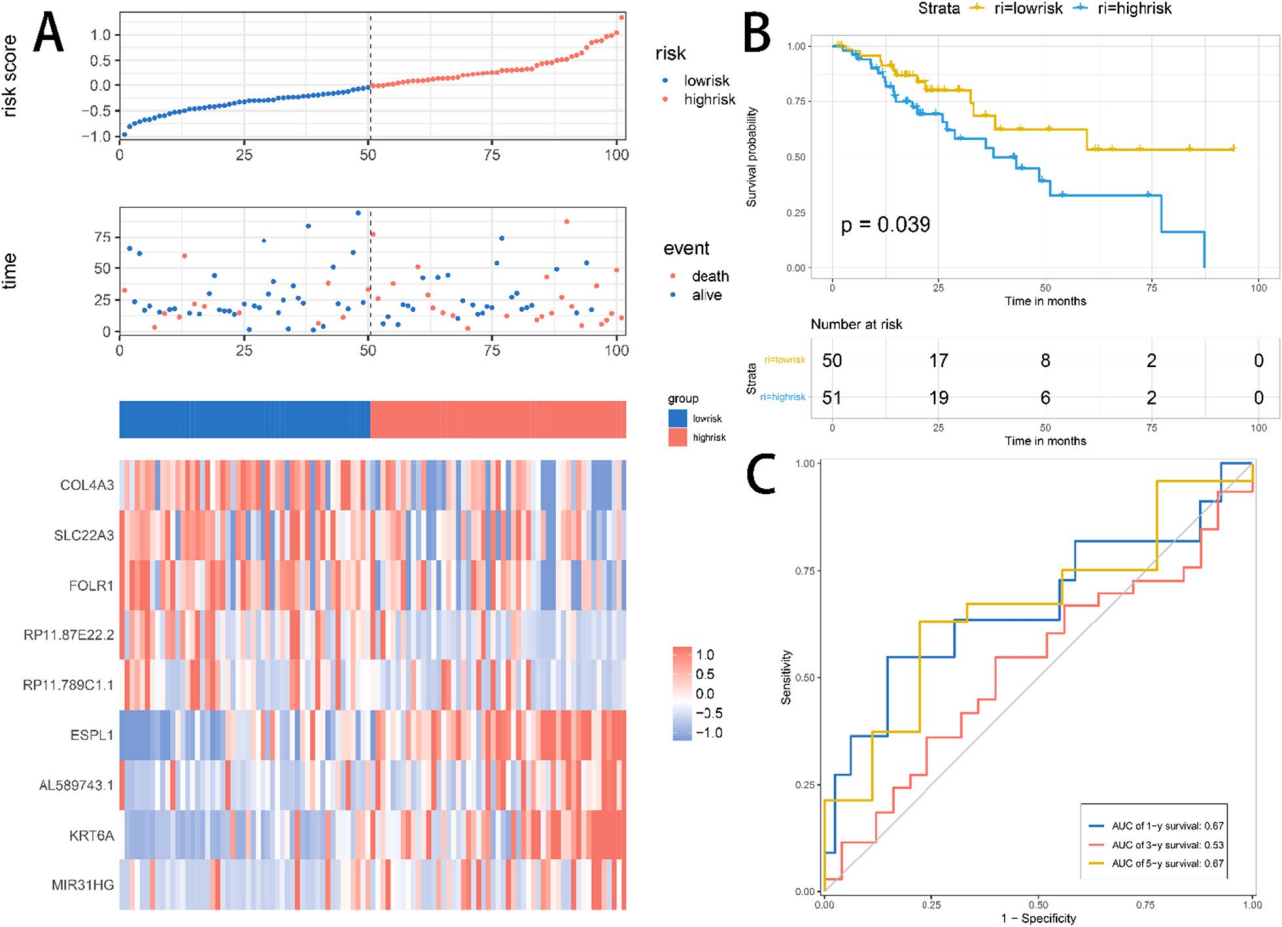
(**A**) Distribution of risk scores, survival time, and heatmap of expression of 9 genes in the test set. (**B**) The KM survival curve of the risk model in the test set. (**C**) The ROC curve of the risk model in the test set.

Risk score distributions and gene expression heatmap of the test set were shown in Fig. 6A**.** Consistent with the trend in the training set, indicating a better prognosis for samples with low risk scores. The KM survival curve demonstrated a significant prognostic difference between the high-risk and low-risk groups in the test set (Fig. 6B). Figure 6C shows the predictive classification performance based on risk scores at 1, 3, and 5 years.

### Relevance analysis of risk models to clinical characteristics

**Figure 7:**
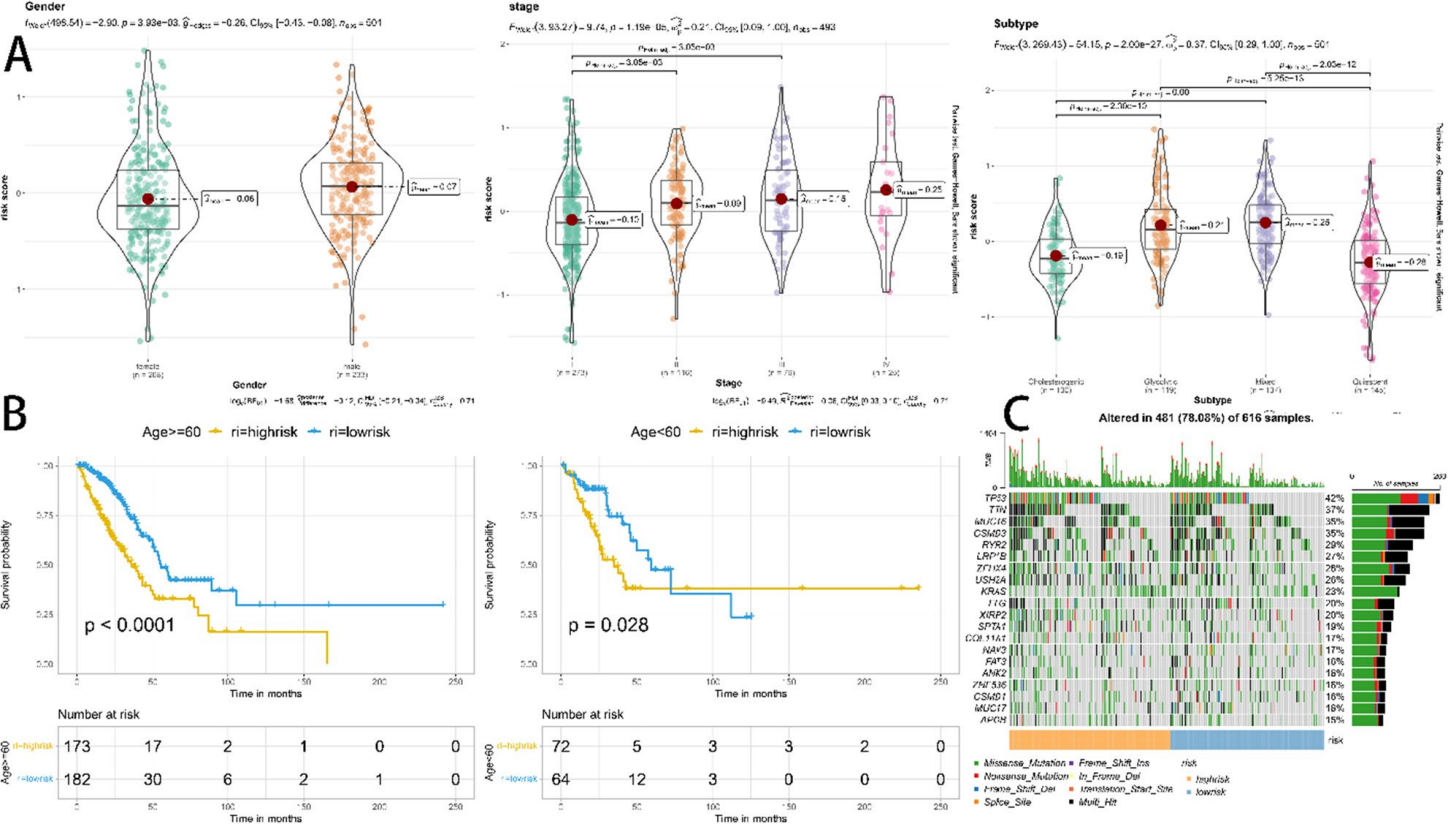
(**A**) Association of risk scores between samples of various gender, stage, and subtype groups. (**B**) Prognosis comparison of various age groups according to risk scores. (**C**) Waterfall plot displaying details of mutations in each gene for each sample in the high and low risk groups.

Risk scores were associated with clinical characteristics significantly (Fig. 7A). By gender, risk scores were significantly higher in the male group than in the female group; by stage, the higher the stage, the higher the risk score; by subtype, risk scores were higher for the mixed subtype with a poor prognosis and lower for the cholesterol subtype with a good prognosis. Therefore, the prognostic model has shown good predictive performance with respect to different clinical characteristics. According to the risk score, age can be classified into high and low risk groups with significant prognostic differences (*p* < 0.05, Fig. 7B). A waterfall plot was used to present mutation data for each gene in high and low risk groupings per sample (Fig. 7C).

### Analysis of the correlation between prognostic genes and TME

**Figure 8:**
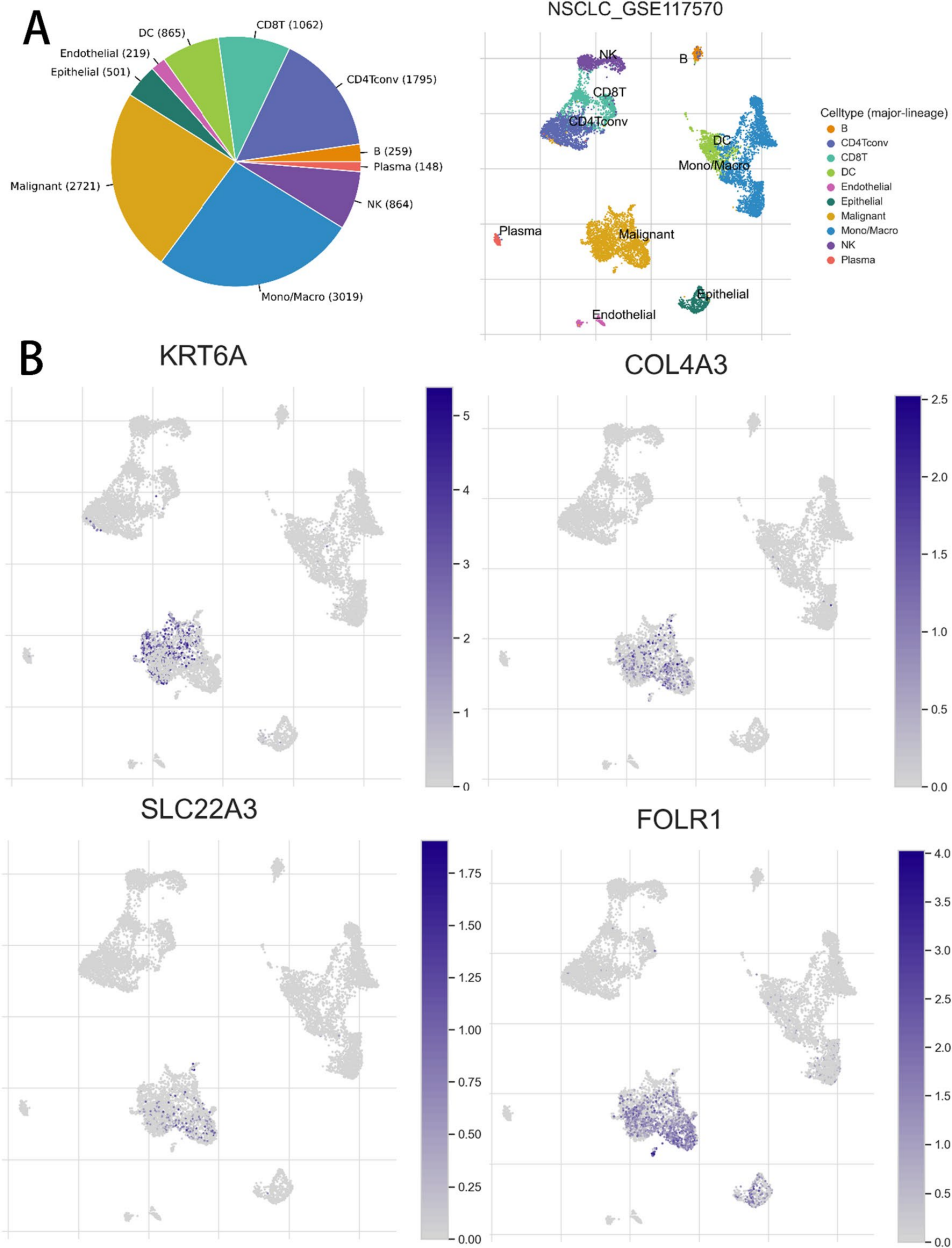
(**A**) Number of cells of each cell type in the NSCLC_GSE117570 dataset, with a description of the distribution of TME-associated cells of each type. (**B**) Distribution of COL4A3, FOLR1, KRT6A, and SLC22A3 in TME-associated cell types.

Given the role of TME in tumor development and its impact on prognosis, we used a NSCLC_GSE117570 dataset from the TISCH database to analyze the expression of some prognostic genes in TME-associated cells. We then examined the dataset, which is categorized into ten cell types. Figure 8A shows the number of cells of each cell type and presents the distribution of each type of TME-associated cells. In this dataset, malignant cells were the most abundant (n = 2721). We found that COL4A3, FOLR1, KRT6A, and SLC22A3 had higher expression in malignant cells compared to other types of TME-associated cells (Fig. 8B). These results support the association of prognostic genes with lung cancer.

## Discussion

Lung cancer is one of the most deadly malignancies in humans, and most patients with advanced lung cancer experience recurrence and treatment resistance. The abnormal metabolism of cancer cells characterized by high glycolysis that occurs even in the presence of high amounts of oxygen, a metabolic reprogramming called the Warburg effect or aerobic glycolysis, has been recognized as a new hallmark of cancer^34^. Inhibition of glycolysis is considered a therapeutic option for aggressive cancers, including lung cancer, and related genes can be used as potential targets for metabolic therapy against cancer cells, such as ARID1A and circ-ENO1^35–37^. Altered metabolism is not limited to cellular energy pathways but also includes alterations in lipid biosynthesis and other pathways (e.g., polyamine processing) in lung cancer and can affect its surrounding microenvironment^38^. It has been shown that lung cancer tissues demonstrate elevated cholesterol levels because the proliferation of cancer cells depends heavily on its availability. Strategies to reduce cholesterol synthesis or inhibit cholesterol uptake have been proposed as potential antineoplastic therapies^39,40^. Therefore, it is essential to clarify the metabolic pathways of lung cancer for its prevention and treatment.

In this study, based on 93 glycolysis and cholesterol synthesis genes, in order to find the most representative genes, consistent clustering was used to minimize the gene numbers, yielding 7 cholesterogenesis and glycolysis co-expressed genes, respectively. Based on these genes, the samples were classified into four subtypes: glycolytic, cholesterogenic, quiescent, and mixed. Although cholesterol plays a crucial role in tumors, survival analysis showed that cholesterol subtypes have a better prognosis than other subtypes, and randomized controlled trials could not support a survival benefit through lipid lowering in lung cancer patients, the reasons for which deserve further investigation^41^.

Pyruvate is central to carbohydrate, fat, and amino acid metabolism. Pyruvate is appealing as a therapeutic target against cancer because it promotes respiratory reserve capacity and mitochondrial oxygen consumption, which may contribute to the aggressive disease phenotype^42^. Mitochondrial pyruvate carrier(MPC) is one of the critical enzymes responsible for pyruvate transport and oxidation^11^. Low or absent MPC1 and MPC2 levels lead to metabolic disorders and alterations in tumor metabolism, and their restored expression inhibits tumor growth, invasiveness, metastasis, and stemness^43^. By analyzing the expression of MPC1/2, the results showed that there were significantly different expressions of MPC1/2 among different subtypes of metabolism, suggesting that the MPC complex affects the metabolic pathway and thus participates in the malignant progression of lung cancer by regulating the amount of pyruvate entering the mitochondria.

We identified DEGs between the best and worst prognosis subtypes and performed a functional enrichment analysis. The results showed significant enrichment of DEGs between the mixed and cholesterogenic subtypes in terms of p53 signaling pathways, microRNAs in cancer, and cell cycle. Then we decided to build the model using ML, with DEGs as features and ending events as labels. It performs best in the test set based on XGBoost’s powerful ability to handle complex classification problems. SHAP was then used to select the most important nine features. Then the most important nine features which SHAP selected were used to construct a prognostic model using a multivariate cox regression model. And this makes it possible to combine the excellent classification power of ML with the interpretability of the prognostic model.

Nine prognostic genes were included four non-coding RNA genes (RP11.87E22.2, RP11.789C1.1, AL589743.1, and MIR31HG) and five coding protein genes (COL4A3, SLC22A3, FOLR1, ESPL1, and KRT6A). After reviewing the kinds of literature, we found that the role of many prognostic genes has been studied concerning lung cancer and has been revealed to impact tumorigenesis and progression. Specifically, ESPL1 expression was positively correlated with SHAP values, and high expression of ESPL1 has been previously shown to be associated with poor prognosis in lung cancer by Zhao et al.^44^. Similarly, KRT6A, MIR31HG, and FOLR1 have been found to enhance lung cancer proliferation and may be potential therapeutic targets^45–47^. Analyzing the association between these genes and TME, we discovered that some prognostic genes were highly expressed in malignant cells using a single-cell sequencing database, which contributed to our construction of a better prognostic model. Subsequently, all samples were classified into high and low risk groups, and the clinical characteristics of the different risk groups were analyzed. Consistent results with the training set were observed in this test set. The model was robust on the training and test datasets and had a great predictive performance.

There are also some limitations to this study. Firstly, the performance of our model has not been tested externally, and there are doubts about its availability for large-scale use. Secondly, the biological associations between the selected prognostic genes remain to be investigated, and their biological explanations with prognostic profiles are to be explored. Future experimental verification is needed. Finally, using the median risk score as a cutoff value to classify high and low risk needs to be optimized.

## Conclusions

Patients with LUAD were effectively typed by glycolytic and cholesterogenic genes and were identified as having the worst prognosis in the glycolytic and cholesterogenic enriched gene groups. Prognostic genes selected by the XGboost algorithm and SHAP analysis can be used to analyze patient prognosis. The prognostic models can provide an important basis for clinicians to predict clinical outcomes for patients. Of course, our model also has some challenges. In clinical practice, a large proportion of patients with LUDA do not undergo genetic testing. We hope to design a more popular model in the future.

## Data Availability

A publicly available dataset was analysed in this study. The data sets analyzed during the current study are available in UCSC Xena browser (https://xenabrowser.net/ (accessed on 1 July 2022)) and the Molecular Signatures Database v7.5 (http://www.gsea-msigdb.org/gsea/msigdb/ (accessed on 1 July 2022)).
